# Variability in development of the striped rice borer, *Chilo suppressalis* (Lepidoptera: Pyralidae), due to instar number and last instar duration

**DOI:** 10.1038/srep35231

**Published:** 2016-10-12

**Authors:** Guang-Hua Luo, Jing Yao, Qiong Yang, Zhi-Chun Zhang, Ary A. Hoffmann, Ji-Chao Fang

**Affiliations:** 1 Institute of Plant Protection, Jiangsu Academy of Agricultural Sciences, Nanjing 210014, China; 2 School of BioSciences, Bio 21 Institute, University of Melbourne, Parkville, Vic 3010, Australia

## Abstract

The striped stem borer, *Chilo suppressalis* (Walker), is an important insect pest of rice which shows substantial variation in developmental duration among individuals. This variation is currently poorly characterized but it is important from a control perspective because pesticides can only target early sensitive instars. It is unclear whether there are key stages that determine the length of developmental duration of individuals and/or whether variation in instar number contributes to this variation. In this study, a laboratory population and a population recently established from the field were used to test variation in development time across instar stages. The duration of developmental time of *C. suppressalis* started to diverge from the 5^th^ instar onward. Individuals pupated at the 5^th^, 6^th^, 7^th^ or even 8^th^ instar stage. In both populations, both the instar at which the larva pupated and the duration of the last larval instar stage determined total developmental time of an individual. There was little impact of the developmental time of early instars on total developmental duration or on instar number prior to pupation. Sex influenced the number of instars but not development time within this number. The biological and applied significance of uneven development in *C. suppressalis* are discussed.

In insects, the number of larval moults and the duration of larval instars can be influenced by environmental factors such as temperature, photoperiod, nutrition, and carbon dioxide anaesthesia^1^,^2^,^3^,^4^,^5^,^6^. Instar number can also be changed by the presence of other individuals and by rearing density^7^,^8^. For instance in *Diploptera punctata*, there was a tendency for males reared in isolation to have an additional larval instar^7^, and the same phenomenon was found in the locust *Schistocerca gregaria*^8^.

It is generally thought that these responses reflect a compensatory scenario: adverse conditions lead to additional instars that allow insects to compensate and still reach a critical size for metamorphosis^9^. The notion that a threshold size for metamorphosis is required and linked to developmental duration emerged from research on the tobacco hornworm, *Manduca sexta*^5^,^10^. When 3rd or 4th instar larvae of this species were starved and molted at a subnormal weight, the resulting 5th instar larvae which usually develop into pupae continued to grow and molt, developing into 6th or 7th instar larvae before metamorphosis^5^. Instar transitions in themselves may depend on levels of oxygen available to developing larvae which will depend on tracheal development relative to larval size^11^.

As well as varying with environmental conditions, instar numbers can also vary among insects reared under the same constant conditions^12^, suggesting that there is inherent variation in instar development not necessarily connected to environmental responses. This instar variation may be partly due to sex: sexual size dimorphism is common in insects, and by having a higher number of instars females may be able to develop to a larger size on emergence^9^,^13^. In addition, instar number variation may reflect a genetic polymorphism in cases where variation in instars expressed under constant conditions occurs regardless of sex^12^. Variation in instars may also be unrelated to sex and genetic factors and represent a genetically fixed adaptive strategy; instar variability might serve to produce larvae with a range of emergence times to spread risk associated with emergence under unpredictable unfavorable periods, allowing organisms to hedge their bets^9^,^13^,^14^.

The striped stem borer, *Chilo suppressalis* (Walker), is an important insect pest of rice in China, India, south-east Asia, Iran and southern Europe. Field surveys of *C. suppressalis* populations show irregular ontogeny and overlapping generations in this pest, making it difficult to target pesticides at susceptible newly hatched larvae. In laboratory populations, some *C. suppressalis* exhibit prolonged developmental times, suggesting a high level of inter-individual variation^15^. It is unclear whether this variation is due to key instar stages that determine the length of individual developmental time and/or variation in instar numbers as well as sex.

The purpose of this study was to assess these sources of variability under constant conditions. Under a compensation model^9^, we might expect that shorter development at early instar stages could act as a trigger for an increase in instar number, particularly for females. We also tested for differences in developmental patterns between a population reared and selected in the laboratory for more than two years and a recently-established population. Larvae were separately fed with an artificial diet, and the developmental duration of each instar was scored. We suggest that instar number variability is only partly related to compensatory growth, given that instar number is linked to sex but not to the length of early developmental stages. Variability in larval development may also reflect an adaptive response to environmental unpredictability although this needs to be further tested. The applied significance of the results are discussed.

## Results

### Instar of pupation

The results showed *C. suppressalis* larvae underwent pupation after the 5^th^, 6^th^, and 7^th^ larval instars (Table 1), with only one instance of pupation at the 8^th^ instar (see Supplementary Table S1). The laboratory population (*Ind-P*) pupated mainly at the 5^th^ and 6^th^ instar stage, accounting for 44.5% and 53.6% of the total number tested for this population respectively. The recently collected population (*Fie-P*) pupated mainly at the 6^th^ instar stage, followed by the 5^th^ and 7^th^ instar stages, accounting for 64.8%, 25.7% and 8.6% of the total respectively (Table 1). In both populations, individuals reaching pupae had a sex ratio close to 1:1, but ratios differed between instars. Overall, more males pupated at the 5^th^ instar, while more females pupated at the 6^th^ instar (Table 1). In both populations, the distribution across instars was heterogeneous across sex as assessed by contingency analysis (lab population, G_1_ = 20.44, P < 0.001, based on 5/6 instar only; field population, G_2_ = 8.07, P = 0.017, based on 5/6/7 instars). There was also a significant effect of population overall when combined across the sexes (G_2_ = 11.94, P = 0.002), reflecting a higher incidence of later instars in the field population.

Some individuals developed into 7^th^ or 8^th^ instars, and even 9^th^ instars, but most of these died without pupation. For *Ind-P*, two individual larvae died on day 10 and day 36 of the 7^th^ instar stage. For *Fie-P*, two larvae died at the 7^th^ instar stage on day 17 and day 32; one larva developed into an 8^th^ instar and pupated on day 31; one larva developed into an 8^th^ instar and died on day 26, and another individual developed into a 9^th^ instar without pupation and died on day 20 (see Supplementary Table S1).

### Developmental duration of *Chilo suppressalis* from hatching to emergence

**Figure 1:**
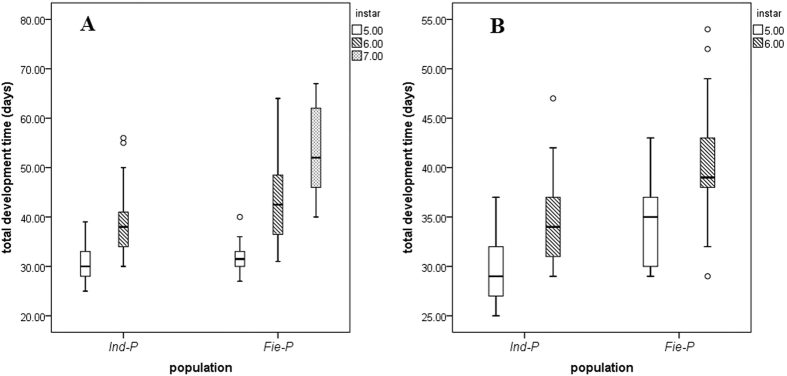
Boxplots of total developmental duration of different instar numbers from the two populations, plotted separately for the two sexes. (**A**) females; (**B**) males. Lines within boxes represent medians, boxes and whiskers represent quartiles.

The developmental duration of each *C. suppressalis* individual is given in Dataset S1 (see Supplementary information). Using a generalized linear model and assuming the data followed a Poisson distribution, we showed that there was a significant effect of instar number (P < 0.001) and population (P < 0.001) on overall development time (Table 2). With increasing instar number, boxplots show that developmental duration increases, and this pattern is apparent for both sexes (Fig. 1). The population difference reflects a shorter development time overall for the laboratory population compared to *Fie-P*. Note that there were no significant sex effects or interactions among the variables. Therefore, within larvae having the same number of instars, the sexes had a similar developmental duration.

For development time of the individual instar stages, the number of instars only had a significant effect on the development time of females at the 5^th^ instar stage, but for males there was also an effect of instar number at the 1^st^ and 2^nd^ instar stages (Table 3: only females that had 5 or 6 instars were considered in these comparisons). Individuals that pupated after 5 instars had a markedly extended development time at the final instar stage when compared to larvae that had 6 and 7 instars. In addition, there was a tendency for males which pupated after 6 instars to have somewhat longer development times in the early instar developmental stages. There were also population differences at early instar stages (Table 3), reflecting the fact that for both sexes the laboratory population had a relatively shorter development period than *Fie-P* in the first two instars but a longer developmental period at the third instar stage. Females that pupated after 6 instars showed an extended development time at this final instar stage in contrast to those that had 7 instars. Moreover, the time spent at the final instar stage increased with the number of larval instars before pupation (Fig. 1).

### Variability in development within classes

We examined the association between total development time and the development time of each instar within sex, population and instar number, to see if the final instar largely controlled variation within as well as between the instar number groups. For females, the five correlations (Spearman) within groups between total development time and the final instar varied from 0.77 to 0.98 and all were highly significant (Table 4). For males, the four correlations involving the final development stage varied from 0.88 to 0.96 and were also all highly significant. In contrast, correlations for the other instar stages were weak and inconsistent in sign (Table 4). These results highlight the strong effect of the final instar stage on the total duration of development and much weaker effects of other stages within instar number groups, a trend that was consistent for the two populations.

This pattern may partly reflect a higher variability in development time at the final instar stage compared to the earlier stages. In order to characterize variability in development time, coefficients of variation (CV) were calculated within instar number groups. These indicate that the CVs of developmental duration from the first instar to the penultimate instar were far lower than those for the last instar, often differing by three fold or more (Table 5). Individuals that pupated after the same number of instars varied in their development at the last instar stage by as much as 20 days (see Supplementary Information Dataset S1).

## Discussion

In this study, we found a high level of variability in developmental duration which was strongly associated with the number of instars at the larval stage as well as variation in the final instar stage before pupation regardless of instar number. Part of the difference in development time due to instar number reflected sex effects. As argued in Esperk *et al*. when females emerge larger they may achieve this by having more instars^13^. While we did not measure the size of emerging moths in this experiment, *C. suppressalis* females are larger than males and we have confirmed this in another colony of this species where we found that females were on average 30% larger than males regardless of the number of instars. Thus the tendency for females to have more instars may partly reflect the fact that they emerge at a larger size, and instar number likely contributes to the development of sexual dimorphism in this species. However, we also found no difference between the sexes in the duration of development once the number of instars had been taken into account. Given that females are larger than males, this points to females also having more efficient growth than males, allowing them to emerge at a larger size even when they have the same instar number.

Although instar number was associated with sex effects, we found it could also vary within sex. This type of variability has been noted for other species, although it is commonly related to external conditions. For instance, in *D. punctata*, all females had 4 larval instars, and males had 3 or 4 instars^7^. In this case, there is a tendency for males reared in isolation to have an additional larval instar and for instar number to vary with other rearing conditions^7^,^16^. In *M. sexta*, instar number is also linked to rearing conditions, and under suboptimal growing conditions growth is slower and larvae achieve final size through a greater number of smaller steps^5^,^17^. However in our work, all *C. suppressalis* individuals were reared in isolation with sufficient food, suggesting that factors other than nutrition are involved.

In other Lepidoptera, variation in larval instars that appears unrelated to sex or external conditions has also been found^12^. For instance, Barraclough *et al*. noted that growth in the geometrid moth, *Pseudocoremia suavis*, fell into two classes depending on instar number (5 or 6) and these classes were independent of sex^14^. In this case, the 6 instar larvae took longer to pupate but had shorter larval durations at the 3–5 instar stage, and changes in instar number likely represented a plastic response to different growth conditions and the need for larvae to reach a particular size for pupation. In contrast, in *C. suppressalis* the extra instars likely lead to an increase in the size of adults rather than involving the attainment of a critical pupation size; in a different colony we found that pupae emerging from larvae with 6 instars were around 12% larger than those emerging from larvae with 5 instars. The absence of substantive differences in development time of individual instar stages (apart from the final stage) between larvae with 5 or 6 instars also suggests that a critical size threshold is not involved in *C. suppressalis*. Instead it appears that variation in instar number (and overall development time) in *C. suppressalis* reflects inherent variation in development time rather than a plastic response to early development.

Insect growth from instar to instar is typically exponential so that most of growth occurs in the last larval instar^18^. Thus, the duration of the last larval instar is often longer than those in all other instars. In *M. sexta*, 90% of mass accumulation occurs in the 5^th^ (last) instar, and the duration of last instar is the longest in all its larval instars^18^,^19^. This probably reflects the need for a final instar larva to feed and grow until it reaches a threshold critical weight, and then undergo changes linked to metamorphosis. In *C. suppressalis*, the average duration of final larval instar is longer than other stages, but the final larval instar duration of each *C. suppressalis* individual varied greatly, as reflected in the high CV values associated with this stage (Table 5). Furthermore, there were significant correlations between the total developmental duration and the last instar duration in individuals in all cases, in contrast to a lack of correlation between total development time and the period between the first instar to the penultimate instar (see Supplementary Table S2). We therefore suspect that mass accumulation rates in each larval instar are different in *C. suppressalis* individuals and that there is variability for critical weight in *C. suppressalis*.

Whether the variability in final instar development time and instar number has a genetic basis or represents a genetically fixed form of bet hedging strategy is unclear. We found instar number variability in both the *Ind-P* and *Fie-P* populations when cultured under the same conditions, despite inadvertent selection in the laboratory population against individuals that showed particularly rapid or slow development during the 15 generations that this population had been cultured; to facilitate maintenance of the *Ind-P* population, individuals that pupated too early or too late were routinely discarded each generation. If development time variation reflected genetic polymorphism, we might have therefore expected lower variability in the *Ind-P* population compared to the *Fie-P* population particularly as laboratory colonies of *C. suppressalis* can lose genetic variation quite rapidly^20^, but this was not reflected in the CVs (Table 5). On the other hand, a genetic shift may have occurred in this population because of the shorter larval duration and lower instar number in the *Ind-P* population.

Because of e variability in larval development, *C. suppressalis* will emerge from pupae across an extended period. In Jiangsu Province, there are two generations of this pest per year. Every year the first generation of *C. suppressalis* larva hatch from the beginning of May to the end of June. The newly hatched *C. suppressalis* larvae are most easily controlled but this extended period of asynchronous hatching makes it difficult to find the most suitable time for application of insecticides. This results in a large amount of insecticides being used to control *C. suppressalis* in the field, likely contributing to the development of resistance to different kinds of insecticides in this pest^21^,^22^,^23^,^24^.

In conclusion, we have shown that *C. suppressalis* has a high level of variability in development linked partly to the number of instars and sex. This variability is expressed under controlled conditions but its biological significance is unclear, apart from obvious effects on sexual dimorphism. One reason why larvae may develop at different rates relates to a tradeoff between size and development: larvae that develop more slowly (and thereby are exposed to an increased risk of parasitism) may nevertheless gain by being larger, leading to a higher reproductive output and mating success^12^. We suggest that two factors other than this tradeoff are also worth exploring in future studies. The first is that larvae show asynchronous development in order to reduce intraspecific competition, both among themselves and also among their offspring. Because female *C. suppressalis* lay eggs in batches, asynchronous development may allow larvae to utilize different resources. The second is that asynchronous development among offspring may represent a response to unpredictable conditions. When confronted by extreme environments, such as postponed planting time of rice due to low temperature, individuals that emergence early may not have access to feeding and breeding sites, whereas late emerging individuals may be at an advantage. However this advantage may be lost in warmer years when early emergence is favored.

## Materials and Methods

### Insects

Two groups of *C. suppressalis* larvae were used in this study: an indoor population (*Ind-P*), which has been cultured in the laboratory for 16 generations, and a field population (*Fie-P*), which was 1st generation larvae acquired newly from the field and propagated in the laboratory. To initiate both the *Ind-P* and *Fie-P* populations, *C. suppressalis* individuals were collected from rice fields in Ruichang County in China. The *C. suppressalis* individuals for the *Ind-P* populations were collected in 2012. The *C. suppressalis* individuals for *Fie-P* populations were collected in 2014. The laboratory line was maintained by turning over at least 500 pupae at the peak of emergence every generation, likely resulting in selection against larvae pupating early or late across multiple generations. Insects were grown at 28 ± 1 °C, with light-dark cycles of 16:8 h and relative humidity of 80%.

### Methods

An artificial diet made in our laboratory was used for development. The diet was made according to Han *et al*.’s method^25^. Medium was cut into small pieces of 1 cm^3^, and placed into six-well plates. Neonate larvae that hatched within 2 hours were transferred into six-well plates with a brush, with one larva per well. A total of 25 plates (150 larvae) were set up. Paper towels were placed between the plate and cover to prevent larvae from escaping.

### Data collection and statistical analysis

The development of each larva was observed every 24 hours, and the molting time, instar stage, pupation time and time of adult emergence were recorded. The total developmental duration refers to the time span from hatching of the egg to adult eclosion.

For analysis, we firstly compared the distribution of the sexes across different number of instar classes by undertaking contingency analysis and computing the G (likelihood ratio) statistic. We also compared the distribution of instars across the two populations with contingency analyses. We then ran a generalized linear model under a Poisson distribution to investigate the impact of population, instar number, and sex on total development time. This trait was not normally distributed even after log (or square root) transformation but did fit a Poisson distribution (Kolmogorov-Smirnov test, P = 0.109). We used boxplots to show the distribution of data in the different groups. We also explored the effects of sex and population on the development period of specific instars through non-parametric comparisons (Kruskal-Wallis tests). Finally, we used non-parametric correlations (Spearman) to investigate the association between instar development and total development within different population and instar number groups. All statistical analyses were performed with SPSS V13.0.

## Additional Information

**How to cite this article**: Luo, G.-H. *et al*. Variability in development of the striped rice borer, *Chilo suppressalis* (Lepidoptera: Pyralidae), due to instar number and last instar duration. *Sci. Rep.* **6**, 35231; doi: 10.1038/srep35231 (2016).

## Supplementary Material

Supplementary Information

Supplementary Dataset 1

## Figures and Tables

**Table 1:** Number of *Ind-P* and *Fie-P* larvae pupating at different instar numbers*.

	5 instars		6 instars		7 instars		Total pupating	
	*Ind-P*	*Fie-P*	*Ind-P*	*Fie-P*	*Ind-P*	*Fie-P*	*Ind-P*	*Fie-P*
Numbers pupating	49	27	59	69	2	9	110	105
% of total	44.5%	25.7%	53.6%	64.8%	1.8%	8.6%	—	—
Females	13	10	41	36	1	8	55	54
Males	36	17	18	33	1	1	55	51
Sex ratio (♀: ♂)	0.36:1	0.59:1	2.28:1	1.09:1	1:1	8:1	1:1	1.06:1

^*^Data included are individuals with successful emergence. “−”: no data.

**Table 2:** Generalized linear model testing for the effects of variables on total development time.

Effect	Wald Chi-Square	df	Significance
Population	16.509	1	<0.001
Sex	1.864	1	0.172
Instar number	84.268	2	<0.001
Population * Sex	1.659	1	0.198
Population * instar number	0.558	2	0.455
Sex * instar number	3.560	2	0.059
Population* sex* instar number	0.557	2	0.455

**Table 3:** Median duration of development (days) for different instar stages when larvae underwent 5, 6 or 7 instars before pupation.

Instar number	Population	Female							Male						
		1^st^	2^nd^	3^rd^	4^th^	5^th^	6^th^	7^th^	1^st^	2^nd^	3^rd^	4^th^	5^th^	6^th^	7^th^
5 instars	*Ind-P*	2 (2,3)	2 (2,5)	3 (3,4)	3 (3,4)	**11 (8,21)**	—	—	2 (2,3)	2 (2,5)	3 (3,4)	3 (2,5)	**10 (7,18)**	—	—
	*Fie-P*	3 (3,4)	3 (3,4)	3 (3,3)	3 (3,4)	**12 (7,20)**	—	—	3 (2,4)	3 (3,4)	3 (3,4)	4 (3,4)	**15 (9,24)**	—	—
6 instars	*Ind-P*	2 (2,3)	3 (2,9)	3 (1,6)	3 (2,7)	4 (2,8)	**14 (5,32)**	—	2 (2,3)	3 (2,4)	3 (2,4)	3 (2,7)	4 (3,5)	**10 (7,25)**	—
	*Fie-P*	3 (2,5)	3 (2,5)	3 (2,5)	3 (2,4)	4 (4,10)	**18 (2,39)**	—	3 (2,4)	3 (2,5)	3 (2,4)	3 (1,6)	4 (3,9)	**16 (8,30)**	—
7 instars	*Ind-P*	—	—	—	—	—	—	—	—	—	—	—	—	—	—
	*Fie-P*	3 (3,4)	5 (2,6)	3 (3,5)	3 (2,10)	4 (3,5)	6 (4,12)	**20 (11,34)**	—	—	—	—	—	—	—
Probabilities (Kruskal-Wallis tests)															
Instar number		0.864	0.126	0.271	0.205	<0.001			0.023	<0.001	0.055	0.119	<0.001		
Population		<0.001	<0.001	0.002	0.488	0.151	0.850		<0.001	<0.001	0.007	0.955	0.068	0.002	

Numbers in brackets represent ranges. Probabilities for Kruskal -Wallis tests comparing instar number and population are given. “—”: no data. Bold numbers represent the last instar.

**Table 4:** Non-parametric correlations between instar development and total development within different population and instar number groups.

	Females					Males			
	5 instars		6 instars		7 instars	5 instars		6 instars	
Larval stage	*Ind-P*	*Fie-P*	*Ind-P*	*Fie-P*	*Fie-P*	*Ind-P*	*Fie-P*	*Ind-P*	*Fie-P*
1st	0.287	0.292	0.306	−0.023	0.000	0.235	−0.142	0.130	0.298
2nd	0.392	−0.525	0.186	0.009	0.096	0.390*	0.155	−0.117	0.083
3rd	0.382	—	0.207	0.043	0.187	−0.038	−0.103	−0.332	0.103
4th	−0.149	0.070	0.198	−0.157	0.082	0.370*	0.073	0.541*	−0.001
5th	0.927***	0.981***	0.097	0.149	0.228	0.891***	0.957***	−0.297	0.093
6th	NA	NA	0.912***	0.962***	0.418	NA	NA	0.878***	0.909***
7th	NA	NA	NA	NA	0.766**	NA	NA	NA	NA
N	13	10	41	36	8	36	17	18	33

^*, **, ***^Correlation is significant at the 0.05, 0.01, 0.001 level respectively. “NA”: not applicable.

**Table 5:** Coefficients of variation for developmental duration from first to penultimate instar, for last instar and for total development time.

Number of instars	Population	Coefficient of Variation (CV) (%)					
		Females			Males		
		1st to penultimate	Last instar	Total	1st to penultimate	Last instar	Total
5 instars	*Ind-P*	8.44	32.98	14.24	10.93	27.21	11.46
	*Fie-P*	3.80	29.94	11.42	3.89	29.81	13.12
6 instars	*Ind-P*	10.20	35.10	15.56	10.77	40.76	13.51
	*Fie-P*	10.65	43.52	19.56	12.87	31.60	14.20
7 instars	*Ind-P*	—	—	—	—	—	—
	*Fie-P*	22.71	39.20	18.55	—	—	—

“−”: no data.
