# Supplementary material for: Variability in development of the striped rice borer, Chilo suppressalis (Lepidoptera: Pyralidae), due to instar number and last instar duration
Source: Sci Rep. 2016 Oct 12;6:35231. doi: 10.1038/srep35231 (PMC5059691; doi:10.1038/srep35231)
Supplement: Supplementary Information [file srep35231-s1.pdf]

Variability in development of the striped rice borer, *Chilo suppressalis* (Lepidoptera: Pyralidae), due to instar number and last instar duration

Guang-Hua Luo *, Jing Yao, Qiong Yang, Zhi-Chun Zhang, Ary A. Hoffmann, Ji-Chao Fang*

Table S1 The developmental durations of some special *C. suppressalis* individuals

Populations		Developmental durations of each instar (days) *									Finally
		1st	2nd	3rd	4th	5th	6th	7th	8th	9th	
Fie-P	Individual 1	3	5	4	3	4	4	14	31	-	Pupated at 8th on day 31
	Individual 2	3	3	4	4	4	15	17	12	20	Died on day 20 at 9th
	Individual 3	3	3	3	4	4	12	10	26	-	Died on day 26 at 8th
	Individual 4	2	4	3	3	2	2	32	-	-	Died on day 32 at 7th
	Individual 5	3	5	2	3	4	3	17	-	-	Died on day 17 at 7th
Ind-P	Individual 1	3	3	6	4	5	6	36	-	-	Died on day 36 at 7th
	Individual 2	3	2	3	3	7	13	10	-	-	Died on day 10 at 7th

"-" stands for no data. Data in shadow are the developmental durations in the last instar.

Table S2 The correlation of the duration between the sum to the penultimate instar from the first instar and the total development process

Instar of pupation	Population	Sex	Pearson Correlation Coefficients	P-value
5th Instar	*Ind-P*	♀	0.332	P=0.268
		♂	0.466	P=0.004
	Fie-P	♀	-0.044	P=0.904
		♂	-0.071	P=0.786
6th Instar	*Ind-P*	♀	0.402	P=0.009
		♂	-0.036	P=0.886
	Fie-P	♀	-0.015	P=0.932
		♂	0.353	P=0.044
7th Instar	*Ind-P*	♀	-	-
		♂	-	-
	Fie-P	♀	0.540	P=0.167
		♂	-	-

"-" stands for no data.
